# Serologic Evidence of Powassan Virus Infection in Patients with Suspected Lyme Disease^1^

**DOI:** 10.3201/eid2308.161971

**Published:** 2017-08

**Authors:** Holly M. Frost, Anna M. Schotthoefer, Angela M. Thomm, Alan P. Dupuis, Sue C. Kehl, Laura D. Kramer, Thomas R. Fritsche, Yvette A. Harrington, Konstance K. Knox

**Affiliations:** Marshfield Clinic Research Foundation, Minocqua, Wisconsin, USA (H.M. Frost);; Marshfield Clinic Research Foundation, Marshfield, Wisconsin, USA (H.M. Frost, A.M. Schotthoefer, T.R. Fritsche);; Coppe Laboratories, Waukesha, Wisconsin, USA (A.M. Thomm, Y.A. Harrington, K.K. Knox);; New York State Department of Health, Slingerlands, New York, USA (A.P. Dupuis II, L.D. Kramer);; Medical College of Wisconsin, Milwaukee, Wisconsin, USA (S.C. Kehl)

**Keywords:** encephalitis viruses, tick-borne encephalitis, tickborne, Powassan virus, deer tick virus, Lyme disease, viruses, zoonoses, vector-borne infections, meningitis/encephalitis, United States, serology

## Abstract

Powassan virus (POWV) lineage II is an emerging tickborne flavivirus with an unknown seroprevalence in humans. In a Lyme disease–endemic area, we examined the seroreactivity to POWV in 2 patient cohorts and described the clinical features of the POWV-seroreactive patients. POWV disease might be less neuroinvasive than previously thought.

Powassan virus (POWV) lineage II, also known as deer tick virus, is an emerging tickborne flavivirus (*1*) transmitted by *Ixodes scapularis* ticks, which are also the primary vector for *Borrelia burgdorferi* (Lyme disease pathogen). In POWV-endemic regions, up to 7% of ticks carry the virus, and seroprevalence among small mammalian hosts can exceed 90% (*2*,*3*). Because the territory of *I. scapularis* is expanding and the prevalence of POWV in ticks and mammals is increasing, POWV poses an increasing threat (*2*–*5*). The seroprevalence of POWV in humans in some regions of North America is known (range 0.5%–3.3%), but because the geographic distribution is quite extensive, the seroprevalence of most at-risk populations is uncertain (*6*). 

POWV is typically detected with an IgM antibody capture ELISA or an IgM immunofluorescence antibody (IFA) assay. Cases are confirmed by >90% or >50% plaque reduction neutralization test (PRNT_90_ or PRNT_50_), detection of virus-specific nucleic acids, isolation in culture, or a >4-fold increase in antibody titers from paired acute and convalescent sera (*7*–*9*). Using these assays, investigators have identified ≈100 cases of POWV encephalitis; however, the actual incidence is likely higher (*1*,*6*). Although nonneuroinvasive disease has been described for other arboviral illnesses, our knowledge of POWV has been limited to patients with neuroinvasive disease (*1*,*8*,*10*,*11*). In this study, we evaluated the seroreactivity for POWV in US Midwest patients, many of whom did not have neuroinvasive disease.

## The Study

We selected patients with suspected tickborne disease (TBD; n = 95) and patients undergoing routine chemical screening (n = 50) who sought treatment during July–August 2015 at the Marshfield Clinic in northern Wisconsin, a TBD-endemic area. Patients were considered to have suspected TBD if a serologic test for *B. burgdorferi* was ordered. The chemical screening cohort included patients who had a complete metabolic or lipid panel ordered as part of their clinical care. We evaluated POWV seroreactivity of specimens from these patient cohorts and, of the patients with serologic evidence of POWV infection and available clinical data, described the clinical features of their disease. All human subject research protocols were approved by the Marshfield Clinic Research Institute Institutional Review Board.

We performed screening assays on all specimens for tick-borne encephalitis virus complex (TBEV-C) and *B. burgdorferi* and performed POWV serology on TBEV-C–positive specimens (Figure; detailed methods in Technical Appendix). To evaluate heterologous flavivirus cross-reactivity, we performed the West Nile virus (WNV) enzyme immunoassay (EUROIMMU, Mountain Lakes, NJ, USA) with TBEV-C–positive samples. We also performed the Flavivirus Mosaic Panel (EUROIMMUN), an IgG IFA assay panel including tests for TBEV, WNV, yellow fever virus, dengue viruses 1–4, and Japanese encephalitis virus, on samples positive for POWV IgG by the IFA assay. Patient vaccination status and travel history were also considered.

**Figure:**
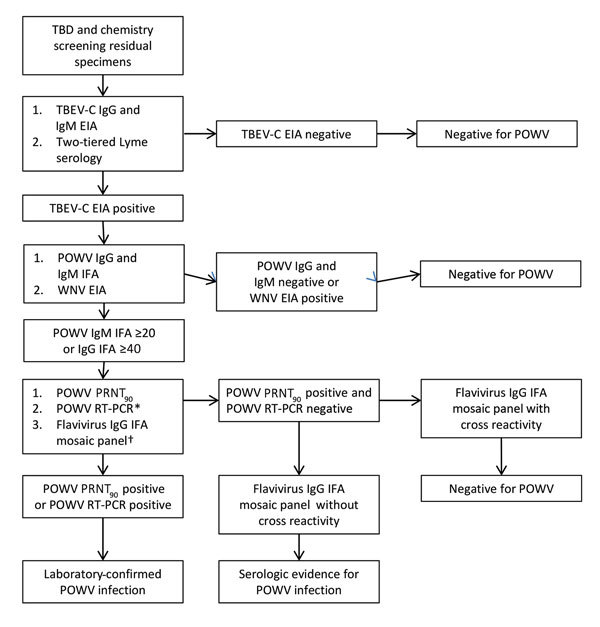
Flow chart showing series of tests performed on specimens obtained from patients with suspected TBD and patients undergoing routine chemical screening to determine POWV seroreactivity, Wisconsin, July–August 2015. *Performed for TBD samples positive for POWV IgG or IgM and chemical screening samples positive for POWV IgM by IFA assay. †Performed for samples positive for POWV IgG by IFA assay. EIA, enzyme immunoassay; IFA, immunofluorescence antibody assay; POWV, Powassan virus; PRNT_90_, >90% plaque reduction neutralization test; RT-PCR, reverse transcription PCR; TBD, tickborne disease; TBEV-C, tick-borne encephalitis virus complex; WNV, West Nile virus.

Clinical data were available for 51 (53.7%) TBD patients and 50 (100%) patients tested by chemical screening with routine chemistry screening completed. For those with clinical data available, we classified their cases as probable or confirmed by using the Centers for Disease Control and Prevention case definitions (*7*). We performed statistical analysis with SAS 9.3 (SAS Institute, Inc., Cary NC, USA) and compared categorical variables by using Fisher exact tests. Significance was defined as p<0.05.

Serologic evidence of POWV infection was present in 9 (9.5%) TBD patients and 2 (4.0%) patients with routine chemistry screening completed (p = 0.33) (Table 1). POWV infection was confirmed in 3 (3.2%) TBD patients (2 by PRNT_90_ [titer range 1:160–1:320] and 1 by reverse transcription PCR) and 0 chemical screening patients (p = 0.55). Of the 3 patients with confirmed POWV infection, evidence of acute infection (IgM positivity) was found in 2 (2.7%). Patients positive only for IgM by IFA assay did not have PRNT_90_ titers, which was expected because neutralizing antibodies are often not present during early infection (*12*). The 2 patients with routine chemistry screening completed who were positive for POWV IgG failed to show neutralization by PRNT; however, rather than PRNT_50_, we used POWV PRNT_90_, which has greater specificity but lower sensitivity. In addition, our PRNT was based on POWV lineage I; thus, our test was potentially less sensitive at detecting POWV lineage II–specific antibodies and thus less capable of detecting previous POWV lineage II infection.

**Table 1:** TBEV-C and *Borrelia burgdorferi* serologic test results and POWV RT-PCR test results of patients with positive POWV IFA assay results, Wisconsin, July–August 2015*

Patient no.	TBEV-C IgM EIA	TBEV-C IgG EIA	POWV IgM IFA assay†	POWV IgG IFA assay‡	POWV PRNT§	POWV RT-PCR¶	*B. burgdorferi*#
Suspected TBD patients							
1**††	–	+	–	+	–	–	–
2††	–	+	–	+	**+**	–	IgG and IgM
3††	+	–	+	–	–	–	IgG and IgM
4††	+	–	+	–	–	–	IgG and IgM
5	+	–	+	–	–	+	–
6	+	–	+	–	–	–	IgG and IgM
7	+	–	+	–	–	–	IgM
8	+	+	+	+	**+**	–	IgG and IgM
9††	+	–	+	–	–	–	IgG and IgM
Patients screened by chemical methods							
1c	+	–	–	+	–	NA	–
2c††	+	+	+	+	–	NA	–

*EIA, enzyme immunoassay; IFA, immunofluorescence antibody; NA, not assayed; POWV, Powassan virus; PRNT, plaque reduction neutralization test; PRNT_90_, >90% plaque reduction neutralization test; RT-PCR, reverse transcription PCR; TBD, tickborne disease; TBEV-C, tick-borne encephalitis virus complex. †Titers >1:20 were considered positive. ‡Titers >1:40 were considered positive. §Positive if sample had a PRNT_90_ titer. ¶Not performed in specimens with a negative POWV IgM IFA assay result. #Samples were screened by EIA and followed up by Western blot. **Cross-reactivity on POWV IgG IFA assay is consistent with a history of West Nile virus infection. ††Clinical data were available.

Similar to other flavivirus serologic assays, considerable cross-reactivity occurred with the Flavivirus Mosaic IgG IFA assay (Technical Appendix Table) (*13*). The fluorescence intensity was stronger for TBEV than it was for other flaviviruses in all TBD patients except for 1 patient with prior confirmed WNV infection. Both patients with routine chemistry screening completed who were POWV IgG–positive were TBEV IgM–positive. Neither had a history of yellow fever or dengue virus exposure or vaccination, although the panel showed cross-reactivity with these viruses.

Evidence of current or prior *B. burgdorferi* infection was present in 63 (66.3%) TBD patients and 4 (8%) patients with routine chemistry screening completed (p<0.0001). Of the 41 (43.2%) TBD patients with evidence of *B. burgdorferi* infection, 7 (17.1%) had serologic evidence of acute POWV infection and 3 (7.3%) had laboratory-confirmed POWV infection. When controlling for differences in seroprevalence rates of *B. burgdorferi*, no statistical differences were evident for POWV seroprevalence (p = 1.0) or confirmed infections (p = 1.0) between patients with routine chemistry screening completed and TBD patients, although the study was underpowered in this regard.

*B. burgdorferi* IgM was detected in 6 (85.7%) of the 7 patients with serologic evidence of acute POWV infection, suggesting concurrent infection, which is consistent with surveillance data indicating that POWV and *B. burgdorferi* co-infect *I. scapularis* ticks (*2*,*3*). The rate of concurrent antibodies we report is higher than that described for regions of Europe endemic for TBE and Lyme disease (*14*).

Clinical data were available for 7 of the patients with serologic evidence of POWV infection (Table 2). Infection probably occurred in 3 patients. A laboratory-confirmed nonacute infection was found in a patient (patient no. 2) who did not meet Centers for Disease Control and Prevention criteria. Patient symptoms could not be attributed specifically to POWV because all TBD patients with clinical data available were positive for *B. burgdorferi* antibodies, and testing for the possibility of infection with additional endemic tick pathogens was performed for only 2 patients.

**Table 2:** Clinical features and histories of patients with positive POWV IFA assay results, Wisconsin, July–August 2015*

Patient no.	POWV test results	*Borrelia burgdorferi* test results†	Clinical features	Comorbidities	CDC case classification	Travel history	Location of tick exposure‡	Vaccine history§
Suspected TBD patients								
1¶	IgG >1:40	IgG and IgM	56-year-old man with 2-wk history of erythema migrans. Treated with doxycycline for 14 d.	Metabolic syndrome, hypertension, 9 y previous had WNV infection		–	Midwest	–
2	IgG >1:40, PRNT 1:160	IgG and IgM	53-year-old man with 3-d history of urticarial rash, malaise, fever, and fatigue. Patient had chills 3 wks prior that resolved. CBC results: leukocytes 7.3 × 10^9^/L, Hb 13.6 g/dL, Hct 39.9%, Plt count 322 × 10^3^/µL; CRP 3.9 nmol/L. PCR neg for *Anaplasma* sp., *Babesia* sp., and *Ehrlichia muris*. Treated with doxycycline for 21 d with complete resolution of symptoms. No history of neuroinvasive disease or TBD.	Hyperlipidemia		–	–	–
3	IgM >1:20	IgG and IgM	14-year-old girl with 3-d history of urticarial rash. CBC results: leukocytes 8.8 × 10^9^/L, Hb 13.0 g/dL, Hct 40.3%, Plt 393 × 10^3^/µL; CRP 3.6 nmol/L. Treated with doxycycline for 14 d.	None		–	–	–
4	IgM >1:20	IgG and IgM	4-year-old girl with 1-wk history of fever (103°F), listless, headache, fatigue, and maculopapular rash. PCR neg for *Anaplasma* sp., *Babesia* sp., and *Ehrlichia muris*. Treated with amoxicillin for 21 d.	None	Probable	–	–	–
9	IgM >1:20	IgG and IgM	3-year-old girl with 1-wk history of intermittent fever, fussiness, and erythema migrans. After development of an urticarial rash, treatment with cefuroxime was changed to amoxicillin for 21 d.	None	Probable	–	Midwest	–
Patients screened by chemical methods								
1c	IgG >1:40	Neg	68-year-old man with no signs or symptoms of acute infectious disease. No history of neuroinvasive disease or TBD. Died from liver cirrhosis.	Coronary artery disease, liver cirrhosis, end stage renal disease		–	–	–
2c	IgM >1:20, IgG >1:40	Neg	76-year-old woman with 2-d history of fever, chills, and MRSA infection of the right hand. Mild abdominal pain and diarrhea occurred later in course. CBC results: leukocytes 13.7 × 10^9^/L, Hb 9.2 g/dL, Hct 29.7%, Plt 180 × 10^3^/µL; CRP 1.5 nmol/L; Procalcitonin 0.1 µg/L. Received daptomycin for 16 d with full recovery. Currently deceased, unknown cause of death.	Congestive heart failure, rheumatoid arthritis on immune-suppressive medications	Probable	–	–	–

*CBC, complete blood cell count; CDC, Centers for Disease Control and Prevention; CRP, C-reactive protein; Hb, hemoglobin; Hct, hematocrit; IFA, immunofluorescence antibody; MRSA, multidrug-resistant *Staphylococcus aureus*; neg, negative; Plt, platelet; POWV, Powassan virus; PRNT, plaque reduction neutralization test; WNV, West Nile virus; TBD, tickborne disease; –, no history. †Samples were screened by EIA and followed up by Western blot. ‡Patient-reported tick exposure. §Known history of vaccination against yellow fever virus, Japanese encephalitis virus, or tick-borne encephalitis virus. ¶Cross-reactivity on POWV IgG IFA assay is consistent with a history of West Nile virus infection.

Consistent with previous studies showing increased susceptibility of children to arboviral diseases, 3 patients who might have had POWV infection were children (Table 2) (*15*). Fever was present in all patients with evidence of POWV acute infection; other common symptoms were fatigue, malaise, fussiness, listlessness, and headache. Complete blood cell count and C-reactive protein did not indicate severe infection. Consistent with other arboviral diseases, urticarial or maculopapular rash was documented in 3 patients (*15*). No patients had neuroinvasive disease.

This study had limitations. Similar to other serologic studies, cross-reactivity and prior exposure to POWV cannot be completely excluded in serologically positive cases. Analysis for other flaviviruses, prior yellow fever virus vaccination, and history of travel to dengue-endemic regions, as well as PRNT, were completed to address this concern. The study population was limited to persons in the US upper Midwest, although POWV is likely an increasing problem throughout the territory *I. scapularis* ticks occupy. Our study results might not be applicable to these other regions.

## Conclusions

In a Lyme disease–endemic area, POWV seroreactivity and confirmed POWV infection were present. The spectrum of disease is broader than previously realized, with most patients having minimally symptomatic infection (*1*,*10*,*11*). Further studies are needed to characterize clinical disease of POWV monoinfection, document POWV seroprevalence in humans, and monitor epidemiologic trends.

Technical Appendix Description of tick-borne encephalitis virus, *Borrelia burgdorferi*, and Powassan virus serologic tests. Flavivirus Mosaic Panel IgG immunofluorescence antibody assay results of patients positive for Powassan virus IgG by the immunofluorescence antibody assay.
