# Supplementary material for: Serologic Evidence of Powassan Virus Infection in Patients with Suspected Lyme Disease
Source: Emerg Infect Dis. 2017 Aug;23(8):1384–8. doi: 10.3201/eid2308.161971 (PMC5547799; doi:10.3201/eid2308.161971)
Supplement: Technical Appendix — Description of tick-borne encephalitis virus, Borrelia burgdorferi, and Powassan virus serologic tests. Flavivirus Mosaic Panel IgG immunofluorescence antibody assay results of patients positive for Powassan virus IgG by the immunofluorescence antibody assay. [file 16-1971-Techapp-s1.pdf]

Article DOI: https://doi.org/10.3201/eid2308.161971

Serologic Evidence of Powassan Virus Infection in Patients with Suspected Lyme Disease

Technical Appendix

On all specimens, we performed screening tick-borne encephalitis virus complex IgM and IgG enzyme immunoassays (EUROIMMUN, Mountain Lakes, NJ, USA) and an in-house, 2-tiered Lyme serologic analysis that included an enzyme immunoassay followed by an IgM and IgG immunoblot on enzyme immunoassay–positive specimens per Centers for Disease Control and Prevention recommendations. Samples with positive tick-borne encephalitis virus complex enzyme immunoassays results were followed up by using Powassan virus (POWV) IgG and IgM immunofluorescence antibody (IFA) assays (Coppe Laboratories, Waukesha, WI, USA) (*1*). The screening dilutions used for the IgM (1:20) and IgG (1:40) POWV IFA assays were based on validation studies (*1*). We validated the POWV IFA assay results with the following specimens: $\geq$90% plaque reduction neutralization test ($PRNT_{90}$)–confirmed, POWV antibody–positive samples provided by the New York State Department of Health; a human heterologous flavivirus sample set (EUROIMMUN; SeraCare, Milford, MA, USA); and yellow fever virus 17D vaccine recipient serum samples (*1*).

Samples identified as having POWV antibodies by IFA assay were sent to the New York State Department of Health for $PRNT_{90}$ testing. All samples positive for POWV IgM and all tickborne disease patient samples positive for IgG were tested by reverse transcription PCR (RT-PCR) assay for the presence of POWV glycoprotein E gene. If positive for E, a confirmatory RT-PCR for deer tick virus nonstructural protein 5 (sense 5′-AACATGATGGGAAAGAGAGAG-3′, antisense 5′-CAGATCCTTCGGTACATGGAA-3′; Coppe Laboratories) was performed (*2*). RT-PCR products were confirmed by gel electrophoresis. Patients were considered to have laboratory-confirmed infection if neutralizing antibodies were found by $PRNT_{90}$ or if POWV-specific nucleic acids were detected (*3*). No paired serum samples were available for evaluation, and virus culture was not performed.

Technical Appendix Table. Flavivirus Mosaic Panel IgG IFA assay results for patients positive for POWV IgG by IFA assay*

	POWV serologic testing		Flavivirus Mosaic Panel IgG IFA assay†								
Patient no.	TBEV-C EIA	POWV IFA assay	TBEV	WNV	JEV	YFV	DENV 1	DENV 2	DENV 3	DENV 4	Uninfected cells
Suspected TBD patients											
1‡	IgG+	IgG+	+	+++	+++	+/–	+/–	++	+	+	–
2‡	IgG+	IgG+	++++	+	+	++	+	++	++	+	–
8	IgG+, IgM+	IgG+, IgM+	++++	++	++++	+++	++	+++	+++	++	–
Chemistry screening patients											
1c‡§	IgG+	IgG+	+	–	–	+	–	+	–	–	–
2c‡§	IgG+	IgG+	+	–	–	+	–	–	–	–	–

*DENV, dengue virus; EIA, enzyme immunoassay; JEV, Japanese encephalitis virus; IFA, immunofluorescence antibody; POWV, Powassan virus; TBD, tickborne disease; TBEV-C, tick-borne encephalitis virus complex; WNV, West Nile virus; YFV, yellow fever virus.
†Number of +'s indicates the degree of immunofluorescent antibody binding.
‡Clinical data were available.
§WNV IgG and IgM EIA were nonreactive.

References

1. Thomm A, Schotthoefer A, Kehr S, Kramer L, Frost H, Fritsche T, et al. Development of a serologic test panel for detection of Powassan virus infection. Post presentation at: The 32st Clinical Virology Symposium; May 19–22, 2016; Dayton Beach, FL, USA. [cited 2017 Feb 12]. http://www.abstractsonline.com/pp8/#!/4039/presentation/725

2. El Khoury MY, Hull RC, Bryant PW, Escuyer KL, St George K, Wong SJ, et al. Diagnosis of acute deer tick virus encephalitis. Clin Infect Dis. 2013;56:e40–7. PubMed http://dx.doi.org/10.1093/cid/cis938

3. Centers for Disease Control and Prevention. Arboviral diseases, neuroinvasive and non-neuroinvasive 2015 case definition [cited 2017 Feb 12]. https://wwwn.cdc.gov/nndss/conditions/arboviral-diseases-neuroinvasive-and-non-neuroinvasive/case-definition/2015/
